# MicroRNAs modulate adaption to multiple abiotic stresses in *Chlamydomonas reinhardtii*

**DOI:** 10.1038/srep38228

**Published:** 2016-12-02

**Authors:** Xiang Gao, Fengge Zhang, Jinlu Hu, Wenkai Cai, Ge Shan, Dongsheng Dai, Kaiyao Huang, Gaohong Wang

**Affiliations:** 1 Key Laboratory of Algal Biology, Institute of Hydrobiology, Chinese Academy of Sciences, Wuhan 430072, China; 2 University of Chinese Academy of Sciences, Beijing 100049, China; 3 School of Life Science, Chinese University of Science and Technology, Hefei 230022, China; 4 Wuxi Biortus Biosciences Co., Ltd., Jiangyin, Jiangsu 214437, China

## Abstract

MicroRNAs play an important role in abiotic stress responses in higher plants and animals, but their role in stress adaptation in algae remains unknown. In this study, the expression of identified and putative miRNAs in *Chlamydomonas reinhardtii* was assessed using quantitative polymerase chain reaction; some of the miRNAs (Cre-miR906-3p) were up-regulated, whereas others (Cre-miR910) were down-regulated when the species was subjected to multiple abiotic stresses. With degradome sequencing data, we also identified ATP4 (the d-subunit of ATP synthase) and NCR2 (NADPH: cytochrome P450 reductase) as one of the several targets of Cre-miR906-3p and Cre-miR910, respectively. Q-PCR data indicated that *ATP4*, which was expressed inversely in relation to Cre-miR906-3p under stress conditions. Overexpressing of Cre-miR906-3p enhanced resistance to multiple stresses; conversely, overexpressing of *ATP4* produced the opposite effect. These data of Q-PCR, degradome sequencing and adaptation of overexpressing lines indicated that Cre-miR906-3p and its target *ATP4* were a part of the same pathway for stress adaptation. We found that Cre-miR910 and its target *NCR2* were also a part of this pathway. Overexpressing of Cre-miR910 decreased, whereas that of *NCR2* increased the adaption to multiple stresses. Our findings suggest that the two classes of miRNAs synergistically mediate stress adaptation in algae.

Epigenetic regulation, especially by means of small RNAs (sRNAs), is involved in the adaptation of plants to environmental stress^1^. Recent studies have shown that miRNAs, the best-characterized class of sRNAs, play essential roles in many biological processes, including development, cell differentiation, growth control, and abiotic and biotic stress resistance^2^,^3^,^4^. Understanding miRNA-guided regulatory networks in growth, development, and stress adaptation can provide new insights for the genetic manipulation of development and stress tolerance^1^. Generation of miRNAs involves formation of the primary miRNA (pri-miRNA) transcripts in the nucleus; these transcripts are then processed by Drosha to yield short precursor miRNAs (pre-miRNAs). The pre-miRNAs are further processed by dicer-1 (DCR-1) in the cytoplasm to yield a duplex containing two strands with miRNA and miRNA^*^. In plants, dicer-like-1 (DCl1) functions in a manner to that of Drosha and Dicer and converts pri-miRNAs to miRNA–miRNA^*^ duplexes. The complementarity between an miRNA and its targeted mRNA determines whether miRNAs modulate gene expression by targeting mRNAs for cleavage or by repressing protein translation^5^. In higher plants, the complementarity of miRNAs and targets is often perfect^6^,^7^ whereas, in animals, extensive mismatches between miRNAs and their targets exists^8^,^9^.

With the development of high-throughput sequencing technologies and the advancement of computational programs, several stress-regulated miRNAs have been identified in model plants subjected to various stresses, including nutrient deficiency^10^, drought^11^,^12^,^13^, cold^14^, salinity^11^,^15^, UV-B radiation^16^, and mechanical stress^1^,^17^. The majority of plant miRNA-related studies have focused on the discovery of miRNAs from different plant species; however, only a few studies have attempted to elucidate the functions of these miRNAs. Therefore, future studies in this field should switch from discovering miRNAs toward validating the roles of individual miRNAs in stress tolerance. Few studies have investigated the common and non-common miRNA-regulated mechanisms that occur during various stresses across various plant species. miRNAs can also be used to alter agriculturally important traits for future crop quality improvement, but related studies are limited.

*Chlamydomonas reinhardtii* is an excellent model organism to understand many basic cell biology functions and the evolutionary transition from unicellularity to multicellularity^18^,^19^. *Chlamydomonas* grow rapidly both photoautotrophically or heterotrophically and are tractable to classical genetic analysis. Sequenced chloroplast^20^ and nuclear genome^21^ allow genomics analysis in this species. Further, expressed sequence tags^22^, microarray information, and other genomic resources are available online. Many studies have investigated the transcription profiles and physiological changes in *Chlamydomonas* subjected to abiotic stress^23^,^24^,^25^,^26^,^27^; RNA interference (RNAi) and CRISPR/Cas9 (clustered regularly interspaced short palindro mic repeats) were successfully applied to *Chlamydomonas* cells^28^,^29^,^30^,^31^,^32^. Therefore, *Chlamydomonas* is a suitable organism for stress biology and is advantageous for conducting research in this field. Previous studies have shown a limited, modulatory role for miRNA-mediated gene regulation in *Chlamydomonas* under standard conditions^6^, but numerous sRNAs are known to be up-regulated under nitrogen or sulfur starvation conditions in wild-type *Chlamydomonas*^33^,^34^,^35^. The findings of these studies suggest that miRNAs might potentially be involved in regulating specific responses to environmental stress rather than in controlling normal cellular functions^36^. Therefore, the regulatory role of miRNAs might be essential to environmental stress adaptation in *Chlamydomonas*. To address these issues, we investigated the expression of miRNAs in *Chlamydomonas* subjected to multiple stresses and identified two types of miRNA expression patterns. The functions of miRNAs under stress were further investigated by using strains overexpressing miRNAs and their target genes; we found that two classes of miRNAs (Cre-miR906–3p and Cre-miR910) played different roles in multiple stress adaptations. We discovered a novel miRNA regulation network that efficiently modulates stress adaptation in *Chlamydomonas*. This study focused on the regulation mechanism of miRNAs in stress adaptation, which is important for stress biology research in plants and algae and might be used to improve algal growth during biofuel^18^ and high-value bio-product^37^ production.

## Results

### Two types of miRNAs are expressed inversely in *Chlamydomonas* under multiple stresses

**Figure 1:**
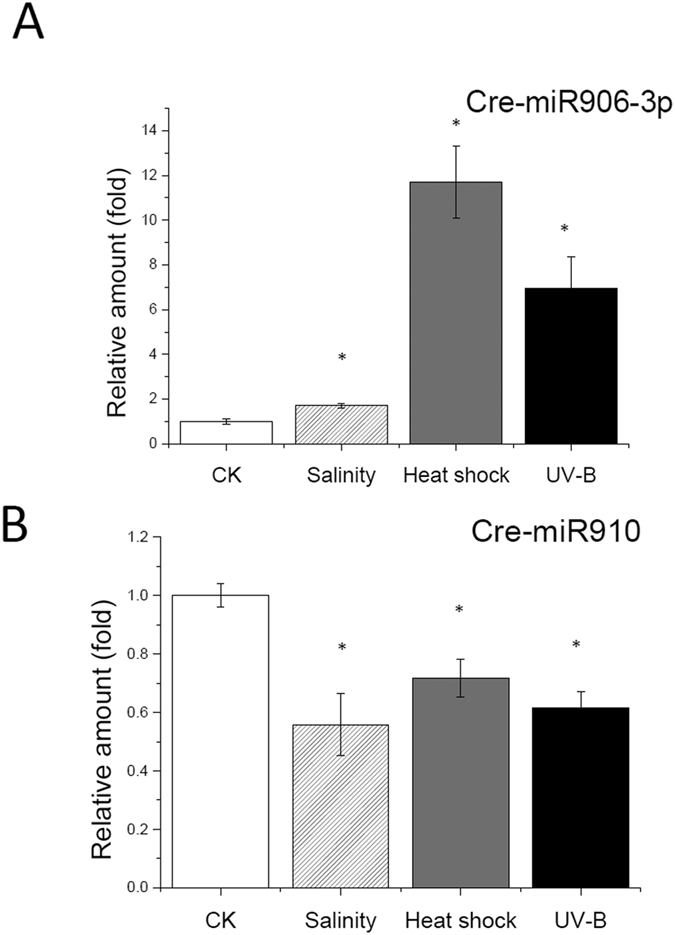
Expression pattern of Cre-miR906-3p (**A**) and Cre-miR10 (**B**) under multiple stresses (heat shock, salinity, and UV-B). *Indicates that differences between the stress treated cells (Heat shock, Salinity and UV-B) and the control cells (CK, no treatment) were considered to be significant at P < 0.05.

Approximately 200 miRNAs have been found in *Chlamydomonas*^33^,^34^; therefore, it is possible to select miRNAs of interest and analyzing their expression pattern under multiple stresses (salinity, heat shock, and UV-B) by using quantitative polymerase chain reaction (q-PCR). We identified 2 classes of miRNAs (Fig. 1) from 8 candidates (Table 1), which exhibited opposite trends in their expression patterns under multiple stresses. The expression of one class (Cre-miR906-3p) was up-regulated, whereas that of the other class (Cre-miR910 and Cre-miR915) was down-regulated under multiple stresses. This result suggested that the two classes of miRNAs potentially play opposite roles in the modulation of stress adaptation in *Chlamydomonas*.

### Prediction of miRNA target genes

The target of each miRNA was predicted using RNAhybrid software (psRNATarget) by searching against the *Chlamydomonas* transcriptome in Phytozome version 9.1^6^,^38^,^39^. We selected Cre-miR906-3p and Cre-miR910 as representatives of the class that was up-regulated and down-regulated, respectively. We identified 6 target mRNAs for Cre-miR906-3p and 5 for Cre-miR 910 (Table 2).

The predicted target genes of Cre-miR906-3p included *ATP4* (ATP synthase subunit D), *Lipase-3* (triglyceride lipase), *CPLD8* (conserved expressed protein with YGGT domain), *NRAMP1* (natural resistance-associated macrophage protein, metal ion transporter, manganese transport protein mntH), *ACH1* (aconitate hydratase), and *RRF* (ribosome recycling factor).

The predicted target genes of Cre-miR910 included *NCR2* (NADPH: cytochrome P450 reductase), *G6PD* (gucose-6-phosphate dehydrogenase), *ACDH* (acyl-CoA dehydrogenase), *ADH* (aldehyde dehydrogenase), and *TRD* (transcription regulator dachshund, contains the SKI/SNO domain).

### Degradome sequencing to identify the corresponding targets of miRNAs

To further identify the targets of Cre-miR906-3p and Cre-miR910, we performed parallel analysis of RNA ends (degradome sequencing) by using RNA samples from the control and NaCl-stressed *Chlamydomonas*^39^. Degradome sequencing can experimentally reveal miRNA targets by using bioinformatic analyses. With a cutoff of two-fold decrease and *P* value of <0.05, in addition to a complementary match in the corresponding 3′ untranslated region with specific microRNAs (details in Materials and Methods), we identified three mRNAs for Cre-miR906-3p and two for Cre-miR910 (Table 3).

The three Cre-miR906-3p targets are *ATP4, CPLD8*, and *ACH1*, and the two Cre-miR910 targets are *NCR2* and *ADH*. All these targets are among the previously predicted targets for Cre-miR906-3p and Cre-miR910. We considered *ATP4* and *NCR2* as representative target genes of Cre-miR906-3p and Cre-miR910, respectively, for further studies.

### q-PCR validation of miRNA expression patterns and related target genes

To validate the results of previous screening experiments, we performed q-PCR to determine the expression pattern of miRNAs and their target genes under multiple stresses of various intensities and exposure times.

UV radiation, at different levels and various exposure times, significantly increased the expression of Cre-miR906-3p, but decreased that of *ATP4* (Fig. S1A,B). Considering that *ATP4* is one of the target genes of Cre-miR906-3p, these results suggested that Cre-miR906-3p regulates the expression of *ATP4* under UV-B radiation. Salinity and heat shock had similar influences on the expression pattern of Cre-miR906-3p and its target gene (*ATP4*) (Fig. S1C–F). Increasing the levels and exposure times of salinity and heat shock treatments concomitantly increased Cre-miR906-3p expression and decreased *ATP4* expression (Fig. S1E,F), except for the samples subjected to heat shock of 50 °C, which showed very low expression levels likely because exposure to such high heat might have killed most of the cells.

Further, we investigated the effects of these stresses on the expression of Cre-miR910 and its target gene *NCR2*. Different levels and exposure times of UV radiation resulted in the down-regulation of Cre-miR910 (Fig. S2A,B), but significant up-regulation of *NCR2*. The influences of heat shock and salinity on the expression pattern of Cre-miR910 and its target gene *NCR2* were similar to those after exposure to UV-B (Fig. S2C–F). Increasing the levels and exposure times of heat shock and salinity resulted in the down-regulation of Cre-miR910 expression and up-regulation of *NCR2* expression (Fig. S2). However, the expression of Cre-miR910 and *NCR2* in the samples subjected to heat shock of 50 °C was very low, as noted for Cre-miR906-3p and *ATP4*.

These results indicated that multiple stresses up-regulated the expression of Cre-miR906-3p and down-regulated that of its target gene, whereas it produced an opposite effect in Cre-miR910 and its target gene.

### Overexpression of Cre-miR906-3p enhanced tolerance to multiple stresses

**Figure 2:**
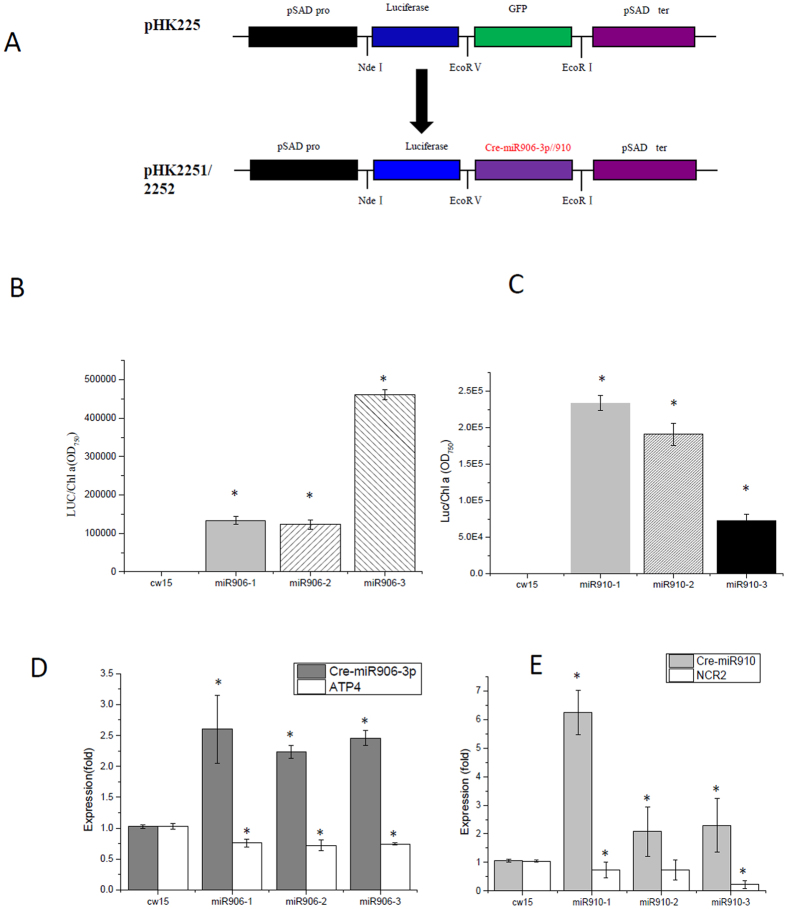
Screening cell lines overexpressing Cre-miR906-3p or Cre-miR910. (**A**) Strategy for the construction of miRNA-overexpressing vector, which includes PsaD promoter (PPsaD), luciferase gene, and Cre-miR906-3p/Cre-miR910 miRNA-overexpressing precursor; (**B**) Screening Cre-miR906-3p-overexpressing cell lines by determining luciferase activity; (**C**) Screening Cre-miR910-overexpressing cell lines by determining luciferase activity; (**D**) q-PCR validation of the expression level of Cre-miR906-3p in the cell lines; (**E**) q-PCR validation of the expression level of Cre-miR910 in the cell lines. The expression folds of miRNA and its target gene were measured in the same samples; *Indicates that differences between the overexpressing lines and the wild-type lines were significant at *P* < 0.05.

**Figure 3:**
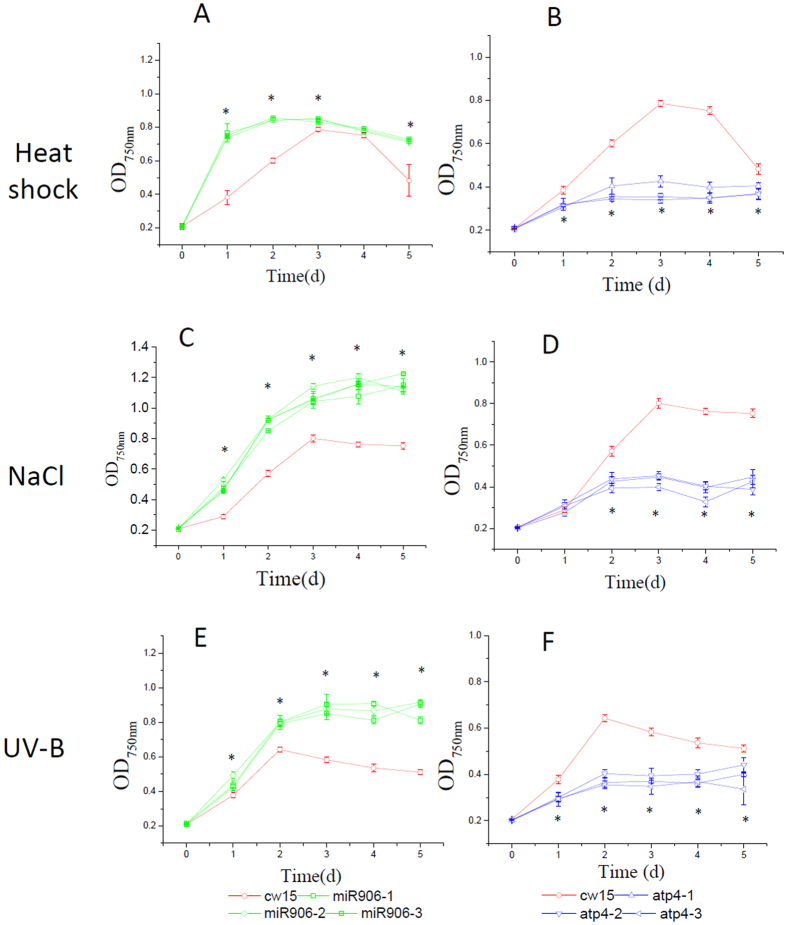
Growth of the cell lines overexpressing Cre-miR906-3p (3 strains represented by green lines and quadrilateral symbols: miR906-1, miR906-2, and miR906-3), *ATP4* (3 strains represented by blue lines and triangle symbols: atp4-1, atp4-2, and atp4-3), and wild-type (cw15, red line with a circle symbol) subjected to multiple stresses. (**A**,**B**) Heat shock; (**C**,**D**) Salinity; (**E**,**F**) UV-B. Technical and biological repeats are 3; *indicates that differences between the overexpression lines (all 3 biological repeats) and the wild-type lines (cw15) were considered to be significant at P < 0.05.

**Figure 4:**
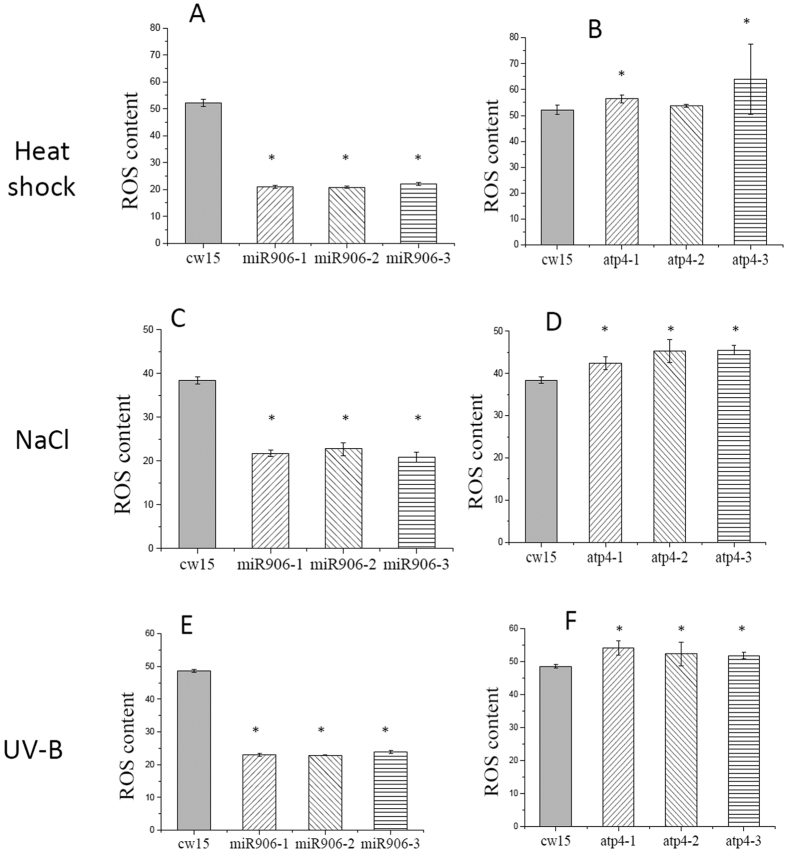
ROS production of the cell lines overexpressing Cre-miR906-3p (3 strains, mir906-1, mir906-2, and mir906-3), *ATP4* (3 strains, atp4-1, atp4-2, and atp4-3), and wild-type (cw15) subjected to multiple stresses for 1 d. (**A**,**B**) Heat shock; (**C**,**D**) Salinity; (**E**), (**F**) UV-B; Technical and biological repeats are 3. *Indicates that differences between the overexpressing lines and the wild-type lines were significant at *P* < 0.05.

The q-PCR results suggested that the expression of Cre-miR906-3p increased under multiple stresses, suggesting that it might be involved in stress adaptation and play positive roles in the protection of cells from circumstantial adversity. To test this hypothesis, we used a previously developed artificial miRNA system to overexpress Cre-miR906-3p in *Chlamydomonas* cells to determine the function of Cre-miR906-3p (Fig. 2A)^19^. More than 3 independent transgenic lines harboring Cre-miR906-3p-overexpressing constructs were selected for further study (Fig. 2B,C; Fig. S3A). Growth experiments showed that the Cre-miR906-3p-overexpressing lines (m906-1, m906-2, and m906-3) grew faster than the wild-type line (cw15) under heat shock (Fig. 3A), salinity (Fig. 3C), and UV-B radiation (Fig. 3E), suggesting that the cells overexpressing Cre-miR906-3p showed higher stress adaptation than the wild-type cells under multiple stresses. Next, we measured the Fv/Fm, which reflects the photosynthetic activity of phototrophic organisms, to determine the photosynthetic activities of the cells under various stresses. The Fv/Fm values of the three Cre-miR906-3p-overexpressing lines were higher than those of the wild-type cells (cw15) under multiple stresses (Fig. S4), implying that cells overexpressing Cre-miR906-3p could maintain higher photosynthetic activity than could the wild-type ones under stress. Subsequently, we compared the mortality rate of cells under stress by using flow-cell analysis. The mortality rates in the three Cre-miR906-3p-overexpressing lines decreased under heat shock (Fig. S5A), salinity (Fig. S5C), and UV-B radiation (Fig. S5E); the number of dead cells was less in Cre-miR906-3p-overexpressing lines than in the wild type under multiple stresses. In addition to the mortality rate, reactive oxygen species (ROS) generation by cells can also reflect the level of damage in cells subjected to stress. In this study, we found that ROS production in the three Cre-miR906-3p-overexpressing lines was lower than that in the wild-type cells (cw15) under multiple stresses (Fig. 4), indicating that Cre-miR906-3p overexpression might protect the cells from stress-related damage. These results suggest that Cre-miR906-3p plays a positive role in stress adaptation and enhances tolerance to multiple stresses.

### Overexpression of the Cre-miR906-3p target gene repressed the tolerance ability to multiple stresses

**Figure 5:**
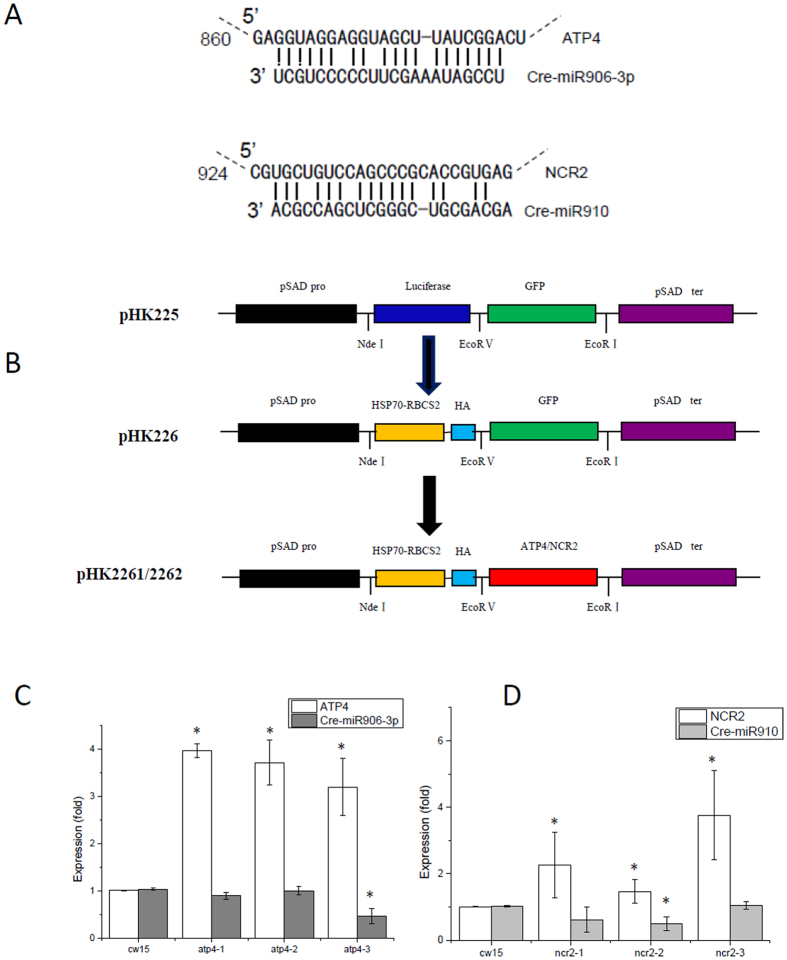
Screening cell lines overexpressing *ATP4* or *NCR2* genes. (**A**) miRNA-mRNA pairing: Cre-miR906-3p and ATP4 (up); Cre-miR910 and NCR2 (down); (**B**) Strategy for the construction of miRNA-overexpressing vector, which includes the *HSP70-RBCS2* promoter, *HA* tail, and *ATP4*/*NCR2* gene; (**C**) q-PCR validation of the expression level of *ATP4* in the cell lines; (**D**) q-PCR validation of the expression level of *NCR2* in the cell lines. The expression folds of miRNA and its target gene were measured in the same samples; *indicates that differences between the overexpressing lines and the wild-type lines were significant at *P* < 0.05.

The best strategy for the functional study of miRNAs is to knock down or knock out miRNA genes. Although knockout mutations with CRISPR-CAS9 are employed to the *Chlamydomonas* cells successfully, low targeting efficiency and complex mutation procedure of this technique limit its application^28^,^40^. Therefore, we adopted an alternative strategy and overexpressed the target gene to counteract the effect of miRNAs (Fig. 5A). miRNAs might function by depressing the translation or degrading of the target genes. When the expression level of target genes far exceeds that of miRNAs, the function of miRNAs will offset. To determine the effect of target gene overexpression, we screened more than 3 cell lines harboring *ATP4* overexpression (Fig. 5B,C; Fig. S3C,E). Growth experiments showed that the three *ATP4*-overexpressing lines (atp4-1, atp4-2, and atp4-3) exhibited lower growth rate than that of the wild-type cells (cw15) under heat shock (Fig. 3B), salinity (Fig. 3D), and UV-B radiation stresses (Fig. 3F). The Fv/Fm values of the 3 ATP4-overexpressing lines were significantly lower than those of the wild-type cells under heat shock, salinity, and UV-B radiation stresses (Fig. S4). Furthermore, the mortality rates of the 3 *ATP4*-overexpressing lines were higher than those of the wild-type cells under multiple stresses (Fig. S5B,D,F). Further, cells overexpressing *ATP4* exhibited higher or similar ROS production to that in the wild-type cells under multiple stresses (Fig. 4). Taken together, these results suggest that cells overexpressing *ATP4* show lower tolerance to multiple stresses; these findings support our hypothesis that the overexpression of Cre-miR906-3p enhances tolerance to multiple stresses, whereas that of the Cre-miR906-3p target gene depresses tolerance to multiple stresses.

### Overexpression of Cre-miR910 depressed tolerance to multiple stresses

**Figure 6:**
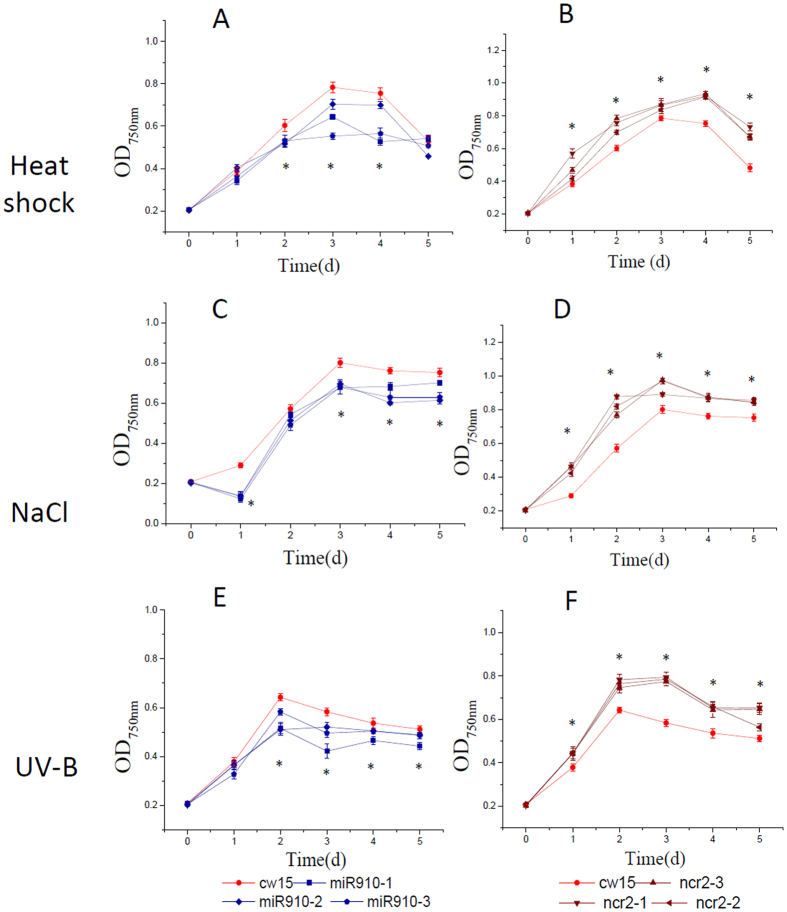
Growth of the cell lines overexpressing Cre-miR910 (3 strains represented by olive lines and quadrilateral symbols: mir910-1, mir910-2, and mir910-3), *NCR2* (3 strains represented by wine lines and triangle symbols: ncr2-1, ncr2-2, and ncr2-3), and wild type (cw15, red line with circle symbol) subjected to multiple stresses. (**A**,**B**) Heat shock; (**C**,**D**) Salinity; (**E**,**F**) UV-B. Technical and biological repeats are 3; *indicates that differences between the overexpression lines (all 3 biological repeats) and the wild-type lines (cw15) were considered to be significant at P < 0.05.

**Figure 7:**
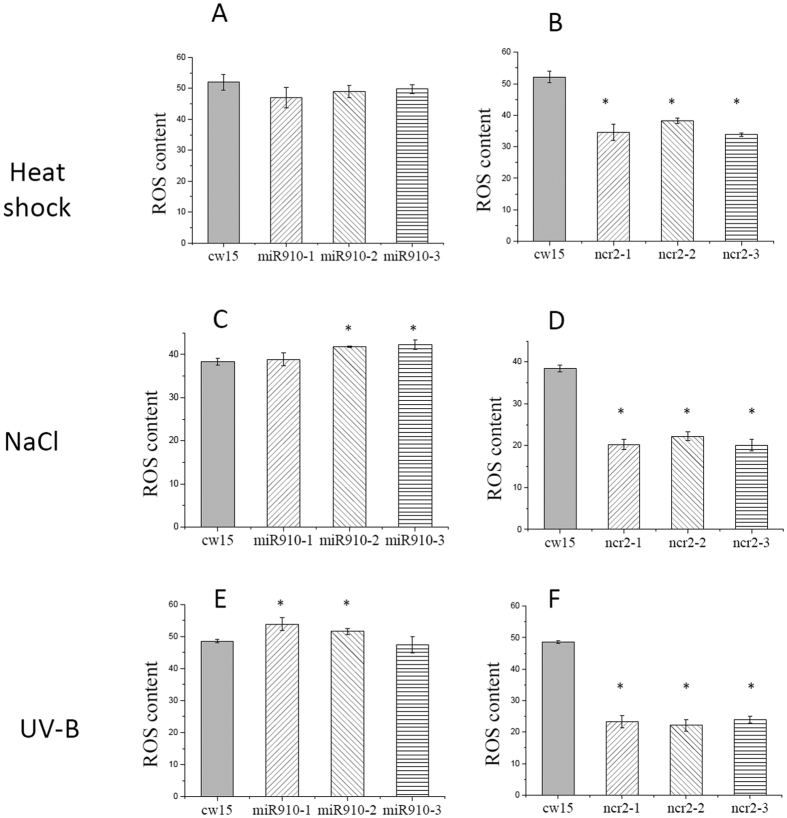
ROS production of the cell lines overexpressing Cre-miR910 (3 strains, miR910-1, miR910-2, and miR910-3), *NCR2* (3 strains, ncr2-1, ncr2-2, and ncr2-3), and wild type (cw15) subjected to multiple stresses. (**A**,**B**) Heat shock; (**C**,**D**) Salinity; (**E**,**F**) UV-B; Technical and biological repeats are 3. *Indicates that differences between the overexpressing lines and wild-type line were significant at *P* < 0.05.

The q-PCR results suggested that Cre-miR910 was down-regulated under multiple stresses, indicating that it plays a negative role in stress adaptation. To test this hypothesis, we selected 3 Cre-miR910-overexpressing cell lines for further study (Fig. 2A,C,E; Fig. S3B). The three Cre-miR10-overexpressing lines (miR910-1, miR910-2, and miR910-3) showed lower growth rate than the wild-type cells (cw15) under multiple stresses (salinity, heat shock, and UV-B radiation; Fig. 6). The Fv/Fm values of Cre-miR910-overexpressing lines declined under multiple stresses (Fig. S6). The mortality rates of these lines increased under multiple stresses (Fig. S7). Both Cre-miR910-overexpressing lines and wild-type cells exhibited high ROS production under multiple stresses (Fig. 7), indicating that both the cell types were damaged by multiple stresses. These results suggested that Cre-miR910-overexpressing cells had lower stress adaptation ability than the wild-type cells, and Cre-miR910 might play a negative role in stress adaptation.

### Overexpression of the Cre-miR910 target gene enhanced tolerance ability to multiple stresses

To determine the function of the miRNA Cre-miR910, we constructed cell lines to overexpress its target gene (*NCR2*) in *Chlamydomonas* (Fig. 5A,C; Fig. S3D,F). We aimed to determine whether the overexpression of the target gene can offset the function of Cre-miR910 in *Chlamydomonas*. The 3 *NCR2*-overexpressing lines (ncr2-1, ncr2-2, and ncr2-3) exhibited a higher growth rate than the wild-type cells (cw15) under multiple stresses (salinity, heat shock, and UV-B radiation; Fig. 6). The Fv/Fm values of the 3 *NCR2*-overexpressing lines (ncr2-1, ncr2-2, and ncr2-3) were higher than those of the wild-type cells under multiple stresses (Fig. S6). In addition, the mortality rates of the 3 cell lines were lower than those of the wild-type cells (Fig. S7). ROS production for the three *NCR2*-overexpressing lines was lower than that in the wild-type cells under multiple stresses (Fig. 7B,D,F). These results suggested that cells overexpressing *NCR2* have a higher tolerance to multiple stresses than the wild-type ones, further supporting our hypothesis that Cre-miR910 plays a negative role in tolerance to multiple stresses.

## Discussion

Over the course of evolution, plants have developed sophisticated mechanisms to cope with the diverse environmental conditions in which they live^1^,^41^. Under optimal conditions, all resources are mobilized to support plant growth and development; however, under stress, growth and development are adversely affected, and resources are used for the adaption process to enable survival^1^. To endure stress, plants need to repress cell division and slow down cell growth, in order to reduce ROS leakage from metabolic pathways and mitigate cell damage. Many stress-upregulated miRNAs are known to be involved in this stress adaptation. Plants have been found to accumulate miR166, miR160, miR393, miR169, miR156, and miR159 under multiple stresses such as UV-B, salinity, cold, and heat stress^1^,^12^. These miRNAs target *TIR1, NF-YA, SBPs*, and *MYBs/TCPs*, the down-regulation of which reduces cell division and growth and enhances survival under biotic- and abiotic-stress conditions^1^,^42^,^43^. This study aimed to determine whether these types of miRNA are present in *Chlamydomonas*. We performed q-PCR screening and found that Cre-miR906-3p was up-regulated under multiple stresses (heat shock, salinity, and UV-B), suggesting that it might play an essential role in a common adaptation mechanism to multiple stresses. We generated cell lines overexpressing Cre-miR906-3p to study its function. Adaptation experiments indicated that the photosynthetic activity and growth were higher, and the mortality rate and ROS production were lower in Cre-miR906-3p-overexpressing strains than in the wild-type strain under stress. This indicated that Cre-miR906-3p-overexpressing strains had higher resistance to multiple stresses (heat shock, salinity, and UV-B) than the wild-type strain. Similarly, miRNAs are known to function by degrading or reducing the translation of target genes. Our computation prediction data revealed that Cre-miR906-3p targets 6 genes in *Chlamydomonas*, including *ATP4, CPLD8, NRAMP1, ACH1, Lipase-3*, and *RRF*. Degradome sequencing can identify miRNA targets experimentally with bioinformatic analyses, and our degradome sequencing data of the control and salinity treated cells showed that there are three Cre-miR906-3p targets (*ATP4, CPLD8*, and *ACH1*) and two Cre-miR910 targets (*NCR2* and *ADH*) (Table 3). We selected on *ATP4* as a representative target gene of Cre-miR906-3p, and *NCR2* as a representative target gene of Cre-miR910 for further studies. *ATP4* encodes the delta subunit of mitochondrial ATP synthase, which serves as a linker between the F_0_ and F_1_ sectors and plays an important role in the regulation of cell metabolism processes^44^. In *Arabidopsis*, down-regulation of *ATP4* by RNAi reduces mitochondrial ATP synthase levels, resulting in growth retardation and gametophyte development deficiencies^45^. Down-regulation of *ATP4* also increased alternative oxidase capacity and led to specific long-term increases in Ala and Gly levels, which might lead to adjustments in mitochondrial metabolism. In cotton, the expression of the ATP synthase delta subunit is significantly up-regulated during fiber cell elongation^46^. In this study, we found that cell lines overexpressing *ATP4* showed lower photosynthetic activity and growth rates, but higher mortality rates and ROS production than the wild-type strains under stress. These results indicated that overexpression of *ATP4* reduces the resistance to multiple stresses (heat shock, salinity, and UV-B). The reason for this phenomenon might be that *ATP4* expression needs to be down-regulated under stress to slow down cell division and growth to restrict ROS leakage. However, *ATP4*-overexpressing cells are unable to slow down their growth and thus cannot adapt to stress conditions. Therefore, modified *ATP4* expression is an essential mechanism for the adaptation regulation to multiple stresses in algae. This result also provides another evidence to support our hypothesis that Cre-miR906-3p up-regulation enhances the tolerance ability to multiple stresses, and it functions in stress adaptation via the down-regulation of *ATP4*.

Under stress, organisms also need to trigger defense mechanisms such as antioxidant system, DNA repair, and damaged protein degradation to protect cells from further harm. Increasing evidence shows that one class of miRNAs is involved in this regulation of defense processes, e.g., miR398, miR393, miR395, and miR168. Stress-down-regulated miR398 targets four genes, including *CSD1* (cytosolic Cu/Zn-SODs), *CSD2* (chloroplast-localized Cu/Zn-SODs), *COX5b-1* (a subunit of the mitochondrial cytochrome c oxidase), and *CCS1* (the copper chaperone for SOD), which are components of the oxidative stress defense system in plants^47^,^48^,^49^. We aimed to determine whether miR398-like miRNAs exist in *Chlamydomonas*, and whether their down-regulation triggers defense mechanisms to protect cells from stress. Our research findings suggested that Cre-miR910 might be a miR398-like miRNA, which is down-regulated under multiple stresses. To determine the function of Cre-miR910, we generated *Chlamydomonas* cell lines overexpressing Cre-miR910. Adaptation experiments showed that the photosynthetic activity and growth were lower, and mortality rate and ROS production were higher in Cre-miR910-overexpressing strains under multiple stresses (heat shock, salinity, and UV-B). These results showed that the overexpression of Cre-miR10 decreases the tolerance ability to multiple stresses, and that Cre-miR10 plays a negative role in stress adaptation. Computation data also showed that cre-miR910 targets 5 genes in *Chlamydomonas*, including *NCR2, G6PDH, ACDH, ADH*, and *TRD*. We selected *NCR2* as the representative target gene of Cre-miR910 to determine the function of Cre-miR910 since degradome sequencing date revealed that *NCR2* is Cre-miR910 target. *NCR* (or *ATR* in *Arabidopsis*) is a membrane-bound flavoprotein and transfers reducing equivalents from NADPH to diverse P450 monooxygenases that participate in a broad range of reactions in plants, including biosynthesis of secondary metabolites, fatty acids, lipids, defense-related chemicals, and plant hormones^50^,^51^,^52^. In *Arabidopsis*, the expression of *NCR* (*ATR2*) was induced by wounding, high visible light, fungal elicitors, and UV light^51^. Mutation of the inducible *Arabidopsis thaliana NCR* (*ATR2*) reduced lignin composition and improved saccharification^50^. *NCR* can also enhance desaturation of membrane lipids, which might affect the membrane integrity and lipid composition of cells and might be involved in the protection of plants and algae against stress^51^. Therefore, *NCR* is an important component of the oxidative stress defense system in plants. In the present study, we found that the photosynthetic activity and growth were higher and the mortality rate and ROS production were lower in *NCR2*-overexpressing strains than in the wild-type strain under multiple stresses. These data indicate that *NCR2* overexpression promotes tolerance to multiple stresses. These results support our hypothesis that Cre-miR910 is the miR398-like miRNA in *Chlamydomonas*, the down-regulation of which triggers the expression of defense system-related genes (*NCR2*) to protect cells from stress-induced damage.

**Figure 8:**
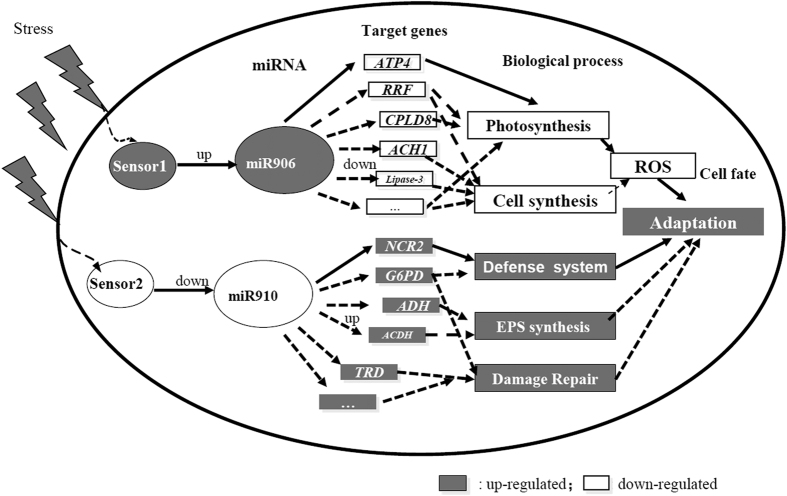
The regulatory network of stress-responsive miRNAs in *Chlamydomonas*. The network is based on the changes in the expression profiles of miRNAs and subsequent target transcripts in algae under stress. Our data support part of this working model that needs further investigations. Gray boxes: up-regulated processes; white boxes: down-regulated processes.

In this study, we described a new miRNA regulatory network in *Chlamydomonas*, which can intricately coordinate biological processes for stress adaptation (Fig. 8). This network comprises two classes of miRNAs. One class was up-regulated under multiple stresses, including Cre-miR906-3p, the up-regulation of which down-regulates the expression of target genes (e.g., *ATP4, Lipase-3, CPLD8, NRAMP1, ACH1*, and *RRF*), and thus reduces ROS production and improves stress adaptation ability. The other class of miRNAs was down-regulated under multiple stresses, such as Cre-miR910, the down-regulation of which induces the target genes (e.g., *NCR2, G6PDH, ACDH, ADH*, and *TRD*) and triggers the activation of the defense system under multiple stresses, leading to the protection of cells from ROS damage and stress. Our study findings might form the basis for understanding the regulatory mechanism of miRNAs in stress adaptation in plants and algae.

## Materials and Methods

### Algal strains, culture conditions, and transformation

The cell wall-defective *C. reinhardtii* strain cw15 (CC-400), kindly provided by *Chlamydomonas* Genetic Center, was used as a recipient strain for all transformations with all miRNAs and for developing the target gene-overexpressing constructs and control construct pHK85^53^, according to the glass bead method^54^. Strains were grown mixotrophically in Tris-acetate-phosphate (TAP) medium^55^ on a rotatory shaker at 25 °C and approximately 25 μE·m^−2^·s^−1^. For transformation, 9 × 10^7^ cells were agitated in the presence of 500 ng plasmids linearized with *Kpn*I and incubated on TAP-agar plates with 10 μg/mL paromomycin. After 7 days of growth on selective medium, transformants were transferred to transparent 96-well-plates containing 200 μL TAP per well, or to 24-well-plates containing 1 mL TAP per well.

### DNA manipulation

The miRNA-overexpressing vectors and target genes were constructed according to our previous method^19^. In brief, the vector pHK225, which carries an *Aph*VIII-selectable marker and the luciferase gene, was used as a backbone to construct miRNA-overexpressing vectors.

The miRNAs were overexpressed by ligating the synthetic sequences of miRNAs precursors (Cre-miR906-3p (Genbank ID: EF497926.1) and Cre-miR910 (Genbank ID: EF495812.1); Supplement 1; for the information on the sequences) into pHK225 (digested with *Eco*RV and *Eco*RI) to create plasmids pHK2251 and pHK2252. The miRNA precursors were established using modified Cre-miR1157 (accession number, MI0006219) to overexpress the miRNAs. For easy screening of the transformants expressing the miRNA, we inserted the luciferase gene from the marine copepod *Gaussia princeps* (G-Luc), the most sensitive bioluminescent reporter expressed in *Chlamydomona*s thus far, upstream of the miRNA precursors (Fig. 2).

The target genes were overexpressed by ligating the sequences of target genes (*ATP4* (Genbank ID: XM_001698684.1) and *NCR2* (Genbank ID: XM_001690078.1); Supplement 1; for the information on the sequences) into pHK226 (digested with *Eco*RV and *Eco*RI) to create plasmids pHK2261 and pHK2262. The luciferase gene of pHK225 was replaced by the 472-bp promoter HSp70A-RBCS2 and a hemagglutinin (HA)-tagged tail to produce plasmids pHK226 (Fig. 5).

### Bioluminescence assays

Luciferase activity was assayed by growing *C. reinhardtii* cultures with miRNA-overexpressing constructs or control constructs in transparent 96-well-plates containing 200 μL liquid TAP per well under constant illumination (approximately 25 μE·m^−2^·s^−1^) to a final cell density of 3–6 × 10^6^ cells·mL^−1^ after three days. After sampling, cells were centrifuged at 10,000 *g* for 1 min at 25 °C. The cell pellet was resuspended in 1 mL phosphate buffered saline (PBS; pH = 7.4) and centrifuged at 10,000 *g* for 1 min at 25 °C to remove the remaining TAP medium. The cells were resuspended in 50 μL lysis buffer following incubation at room temperature for 15 min. The cell lysate was allowed to undergo 2 or 3 freeze-thaw cycles to ensure complete lysis of cells. After thawing, 50 μL of the cell lysate was transferred to 96-well black plates, and 50 μL of the assay buffer was added to each well. Bioluminescence was immediately assayed using a multi-mode microplate reader (Filter Max F5; Molecular Devices). The background was normalized by measuring bioluminescence of wells containing only buffer and those containing buffer with cells having the pHK225-vector.

### Multiple stress treatments

The stress experiments were conducted according to the method of Huma *et al*.^23^ and He & Hader^56^. *Chlamydomonas* cells at the beginning of the stationary phase were used, and their viability was verified. The cells were grown in TAP medium until the density of the cells reached an OD_750_ of 0.2, which is equal to 1 × 10^6^ cells per mL culture.

1. Heat shock: For this condition, 100 mL cell suspensions at the early stationary phase were transferred to 37 °C, 42 °C, and 50 °C water baths and incubated for 15, 30, 45 and 60 min in the dark, respectively. Then, physiological parameters were measured at 25 °C.
2. Salinity: For salinity stress induced using NaCl, the induction stress concentrations were 75, 150, and 225 mM for 1, 2, 3, 4 and 5 d each.
3. UV-B radiation: UV-B radiation was generated from an Ultraviolet-B 40 W tube (Spectroline XX-15A/FA; USA), with its main output at 312 nm and a cellulose acetate filter to screen out UV-C, in addition to regular fluorescent lamps. The UV-B radiation (plus or minus) was measured using a DRC-100X digital radiometer (Spectroline, USA).

Cultures grown in Petri dishes with quartz lids were irradiated with UV-B for different durations (15, 30, 45 and 60 min) and used to determine the photosynthetic activity, growth, morality, and ROS production.

### Degradome sequencing and follow-up bioinformatic analyses

Total RNA was extracted by using TRIzol reagent (Invitrogen, CA, USA) following the manufacturer’s procedure. For each degradome library, 20 μg total RNA was prepared following the method reported by Ma *et al*.^57^. The gene expression level was calculated using R followed with bowtie (–v 1 –m 20 –best) mapping. The *C. reinhardtii* (assembly v3.0) database and transcript sequences were used as reference. All RNA-sequencing data will be deposited in the NCBI Gene Expression Omnibus (GEO) and will be available upon the acceptance of this paper.

### Prediction of microRNA targets

PsRNATarget prediction software (http://plantgrn.noble.org/psRNATarget) was used to predict the targets of microRNAs by using the default parameters of the software^6^,^38^,^39^.

### q-PCR

Cells (10^7^ cells·mL^−1^) were harvested and resuspended in a 1.5 mL RNAse-free micro-tube containing 1 mL TRIZOL reagent (Invitrogen, USA). After precipitation in 100% isopropanol and washing with 75% ethanol, the RNA pellet was suspended in a suitable volume of diethylpyrocarbonate-treated water according to the manufacturer’s instructions. RNA solutions were quantified using NanoDrop Lite (Thermo Fisher., USA). Aliquots were stored at −80 °C.

1. Quantification of miRNA by using qPCR was performed using One-Step PrimeScript^®^ miRNA cDNA Synthesis Kit (D350A, Takara) and miRNA qRT-PCR SYBR^®^ Kit (638314; Takara) according to the manufacturers’ instructions. The forward primers of miRNAs in this study are listed in Table S1, and the reverse primers were included in the kit. U4 was used to normalize miRNA expression data.
2. Quantification of mRNA by using qPCR was performed using PrimeScript RT reagent Kit Perfect Real Time (DRR037S; Takara) and SYBR Premix Ex Taq II (Tli RNaseH Plus; DRR037S; Takara) according to the manufacturers’ instructions. The primers for the target genes used in this study are listed in Table S2. GAPDH was used to normalize mRNA expression data.

### Fv/Fm

The Fv/Fm values were measured at 25 °C by using a PhytoPAM (Walz, Germany). All samples were dark-adapted for 10 min before the measurements.

### Growth

The growth rate was measured at OD_750_ for algae cultures.

### Morality measurement

Flow cytometry with a single 488-nm argon laser was used to measure the cell morality rate after staining with vital dyes. A flow cytometer (Accuri C6; Becton Dickinson, USA) coupled to a propidium iodide (PI) dye (BS078A; Biosharp, China) was used to determine the cell morality of the algae after treatments. PI is a non-fluorescent nuclear DNA staining reagent that is often used for cell apoptosis detection. When it combines with DNA, it can emit a 615 nm red light under a 535 nm wavelength excitation light. It cannot penetrate the membranes of living cells, but can pass through damaged cell membranes and combine with nuclear DNA.

### ROS assay

ROS levels were measured according to the manufacturer’s instructions of a ROS detection kit (Beyotime Institute of Biotechnology, Haimen, China). In this kit, the nonfluorescent probe 2′,7′-dichlorofluorescein diacetate (H2DCF-DA) passively diffuses into cells and is deacetylated by esterases to form nonfluorescent 2′,7′-dichlorofluorescein (DCFH)^56^. In the presence of ROS, DCFH reacts with ROS to form the fluorescent product DCF, which is trapped inside the cells. The fluorescence was read at 485 nm excitation and 530 nm emission by using a fluorescence plate reader (Bio-TEK, USA). The intensity of fluorescence compared with that in the control was considered as the increase of intra-cellular ROS.

### Western blot analysis

For a ~100 mg sample, add ~300 μL of ice-cold lysis buffer rapidly to the tube, then maintain constant agitation for 2 h at 4 °C. Centrifuge for 20 min at 12,000 rpm at 4 °C in a microcentrifuge. Aspirate the supernatant and place in a fresh tube kept on ice; discard the pellet. Remove a small volume of lysate to perform a protein quantification assay. Determine the protein concentration for each cell lysate. Determine how much protein to load and add an equal volume 2× loading sample buffer and boil at 100 °C for 5 min. Load equal amounts of protein into the wells of the SDS-PAGE gel. Then transfer the protein from the gel to the PVDF membrane. Block the membrane for 1 h at room temperature using blocking buffer. Incubate the membrane with 1000× dilutions of Monoclonal Antibody to HA or tubulin (Bio-Transduction Lab, Wuhan) in the blocking buffer. Wash the membrane in three TBST washes, 5 min each. Incubate the membrane with the 1000× dilution of goat anti-mouse IgG-HRP (Bio-Transduction Lab, Wuhan) in the blocking buffer at room temperature for 1 h. For signal development, follow the BeyoECL Star kit (Beyotime biotechnology) manufacturer’s recommendations. Acquire images using darkroom development techniques for chemiluminescence.

### Statistical analysis and Experiment repeats

Data were evaluated using one-way analysis of variance (SPSS-13 for Windows; tests: least significant difference, Tukey’s honestly significant difference). Three technical repeats and 3 biological repeats were applied in all experiments.

## Additional Information

**How to cite this article**: Gao, X. *et al*. MicroRNAs modulate adaption to multiple abiotic stresses in *Chlamydomonas reinhardtii. Sci. Rep.* **6**, 38228; doi: 10.1038/srep38228 (2016).

**Publisher's note:** Springer Nature remains neutral with regard to jurisdictional claims in published maps and institutional affiliations.

## Supplementary Material

Supplementary Information

## Figures and Tables

**Table 1:** MiRNAs expression of *Chlamydomonas* cells under multiple stresses.

miRNAs	CK	Salinity	Heat shock	UV-B	Remarks
Cre-miR919.2	0.998 ± 0.046	0.916 ± 0.272	1.32271 ± 0.566	1.025 ± 0.732	No significant
Cre-miR915	1.000 ± 0.105	0.617 ± 0.221*	0.33143 ± 0.023*	0.526 ± 0.216*	3 down
Cre-miR913-5p	1.001 ± 0.136	1.145 ± 0.249	2.177 ± 1.039*	0.552 ± 0.006*	1down, 1up
Cre-miR908.2	1.000 ± 0.089	0.485 ± 0.445	0.462 ± 0.085*	0.353 ± 0.3111*	2down
Cre-miR1167	0.997 ± 0.024	2.257 ± 0.299*	14.032 ± 6.588*	0.656 ± 0.066*	2 up; 1 down
Cre-miR1152	0.999 ± 0.097	0.781 ± 0.428	0.925 ± 0.334	0.845 ± 0.582	No significant
Cre-miR910	0.999 ± 0.040	0.558 ± 0.107*	0.717 ± 0.066*	0.614 ± 0.057*	3 down
Cre-miR906-3p	1.002 ± 0.103	1.703 ± 0.116*	11.697 ± 1.598*	6.962 ± 1.381*	3 up

Expressions of 8 miRNAs candidates were determined in *Chlamydomonas* cells after exposing to multiple stresses (Heat shock, Salinity and UV-B) with Q-PCR. The values are the ratios of the expression fold of stressed cells to the control cells (CK), and up-or down regulated under all three stresses miRNAs were screened for further study. *Indicates that differences between the stressed cells and the control cells (CK) were considered to be significant at P < 0.05.

**Table 2:** Computation prediction of target genes of miRNAs.

miRNA	Targeted mRNA	Gene Name
Cre-miR910	jgi|Chlre4|116375|e_gwW.1.50.1	NCR2
	jgi|Chlre4|309910|kg.chromosome_8_#_416_#_chlre3.21.61.1.1	G6PDH
	jgi|Chlre4|283132|au.g1636_t1	ADC
	jgi|Chlre4|182952|estExt_fgenesh2_kg.C_100097	TRD
	jgi|Chlre4|128289|estExt_gwp_1 H.C_120106	ACDH
Cre-miR906-3p	jgi|Chlre4|185200|estExt_fgenesh2_kg.C_410003	ATP4
	jgi|Chlre4|152053|Chlre2_kg.scaffold_43000017	Lipase-3
	jgi|Chlre4|154497|Chlre2_kg.scaffold_80000054	YGGT
	jgi|Chlre4|128400|estExt_gwp_1 H.C_130096	NRAMP1
	jgi|Chlre4|129025|estExt_gwp_1 H.C_10164	ACH1
	jgi|Chlre4|303306|kg.chromosome_3_#_229_#_TC48423	RRF

**Table 3:** Identification the corresponding targets of miRNAs by Degradome sequencing.

miRNA	Targets	Fold change	P value
Cre-miR906-3p	CPLD8	0.072	8.45E-04
	ATP4	0.074	1.03E-03
	ACH1	0.288	7.44E-10
Cre-miR910	ADH	2.281	1.51E-07
	NCR2	21.408	1.62E-05
