# Supplementary material for: MicroRNAs modulate adaption to multiple abiotic stresses in Chlamydomonas reinhardtii
Source: Sci Rep. 2016 Dec 2;6:38228. doi: 10.1038/srep38228 (PMC5133633; doi:10.1038/srep38228)
Supplement: Supplementary Information [file srep38228-s1.pdf]

MicroRNAs modulate adaption to multiple abiotic stresses in *Chlamydomonas reinhardtii*

Xiang Gao, Fengge Zhang, Jinlu Hu, Wenkai Cai, Ge Shan, Dongsheng Dai, Kaiyao Huang & Gaohong Wang*

List of supplement files:

1. Figure legends
2. Fig S1
3. Fig S2
4. Fig S3
5. Fig S4
6. Fig S5
7. Fig S6
8. Fig S7
9. Tab S1
10. Tab S2
11. Supplement 1

Figure Legends

Fig. S1 Q-PCR validation of expression pattern of Cre-miR906-3p and its target gene (*ATP4*) under multiple stresses of different levels and exposing times. (A) Expression of Cre-miR906-3p (up) and *ATP4* (down) under UV-B with different levels; (B) Expression of Cre-miR906-3p (up) and *ATP4* (down) under UV-B (100w/m^2) with different treatment times; (C) Expression of Cre-miR906-3p (up) and *ATP4* (down) under salinity (NaCl) with different levels; (D) Expression of Cre-miR906-3p (up) and *ATP4* (down) under salinity (150mM NaCl) with different treatment times; (E) Expression of Cre-miR906-3p (up) and *ATP4* (down) under heat shock with different levels; (F) Expression of Cre-miR906-3p (up) and *ATP4* (down) under heat shock (42 ℃) with different treatment times. * indicates that differences between treated cells and the control (no treatment) cells were considered to be significant at $P < 0.05$.

Fig. S2 Q-PCR validation of expression pattern of Cre-miR910 and its target gene (*NCR2*) under multiple stresses of different levels and exposing times. (A) Expression of Cre-miR910 (up) and *NCR2* (down) under UV-B with different levels; (B) Expression of Cre-miR910 (up) and *NCR2* (down) under UV-B (100w/m^2) with different treatment times; (C) Expression of Cre-miR910 (up) and *NCR2* (down) under salinity (NaCl) with different levels; (D) Expression of Cre-miR910 (up) and *NCR2* (down) under salinity (150mM NaCl) with different treatment times; (E)

Expression of Cre-miR910 (up) and *NCR2* (down) under heat shock with different levels; (F) Expression of Cre-miR910 (up) and *NCR2* (down) under heat shock (42 ℃) with different treatment times. * indicates that differences between treated cells and the control (no treatment) cells were considered to be significant at $P < 0.05$.

Fig. S3 PCR and Western blotting validation of expression miRNAs and target genes in overexpression lines. (A) PCR results for Cre-miR906-3p expression in Cre-miR906-3p overexpression lines (3 strains, named miR906-1, miR906-2 and miR906-3; cw15, wild-typed lines) ; (B) PCR results for Cre-miR910 overexpression in Cre-miR910 overexpression lines (3 strains, named miR910-1, miR910-2 and miR910-3; cw15, wild-typed lines); (C) PCR results for *ATP4* overexpression in *ATP4* overexpression lines (3 strains, named atp4-1, atp4-2 and atp4-3; cw15, wild-typed lines); (D) PCR results for *NCR2* expression in *NCR2* overexpression lines (3 strains, named ncr2-1, ncr2-2 and ncr2-3; cw15, wild-typed lines); (E) Western blotting results for HA tail expression in *ATP4* overexpression lines (3 strains, named atp4-1, atp4-2 and atp4-3; cw15, wild-typed lines); (F) Western blotting results for HA tail expression in *NCR2* overexpression lines (3 strains, named ncr2-1, ncr2-2 and ncr2-3; cw15, wild-typed lines).

Fig. S4 Photosynthesis activity (Fv/Fm) of the cell lines with Cre-miR906-3p overexpression (3 strains with green line and quadrilateral symbol, named miR906-1, miR906-2 and miR906-3), *ATP4* overexpression (3 strains with blue line and triangle

symbol, named atp4-1, atp4-2 and atp4-3) and wild-type (cw15, red line with circle symbol) under multiple stresses. (A) (B) under Heat shock; (C) (D) under Salinity; (E) (F) under UV-B. * indicates that differences between the overexpression lines (all 3 biological repeats) and the wild-type lines (cw15) were considered to be significant at $P < 0.05$.

Fig. S5 Morality rates of the cell lines with Cre-miR906-3p overexpression (3 strains, named miR906-1, miR906-2 and miR906-3), *ATP4* overexpression (3 strains, named atp4-1, atp4-2 and atp4-3) and wild-type (cw15) under multiple stresses after 1d exposing. (A) (B) under Heat shock; (C) (D) under Salinity; (E) (F) under UV-B. * indicates that differences between the overexpression lines and the wild-type lines (cw15) were considered to be significant at $P < 0.05$.

Fig. S6 Photosynthesis activity (Fv/Fm) of the cell lines of overexpression of Cre-miR910 (3 strains with olive line and quadrilateral symbol, named miR910-1, miR910-2 and miR910-3), *NCR2* overexpression (3 strains with wine line and triangle symbol, named ncr2-1, ncr2-2 and ncr2-3) and wild type (cw15, red line with circle symbol) under multiple stresses. (A) (B) under Heat shock; (C) (D) under Salinity; (E) (F) under UV-B. * indicates that differences between the overexpression lines (all 3 biological repeats) and the wild-type lines (cw15) were considered to be significant at $P < 0.05$.

Fig. S7 Morality rate of the cell lines of overexpression of Cre-miR910 (3 strains,

named miR910-1, miR910-2 and miR910-3), *NCR2* overexpression (3 strains, named ncr2-1, ncr2-2 and ncr2-3) and wild type (cw15) under multiple stresses. (A) (B) under Heat shock; (C) (D) under Salinity; (E) (F) under UV-B. * indicates that differences between the overexpression lines and the wild-type lines (cw15) were considered to be significant at $P < 0.05$.

Tab. S1 The miRNAs information and their primers used in this study

Tab. S2 The primers of target genes and reference genes used in this study

Supplement 1: Information of the sequences in this study

Fig S1

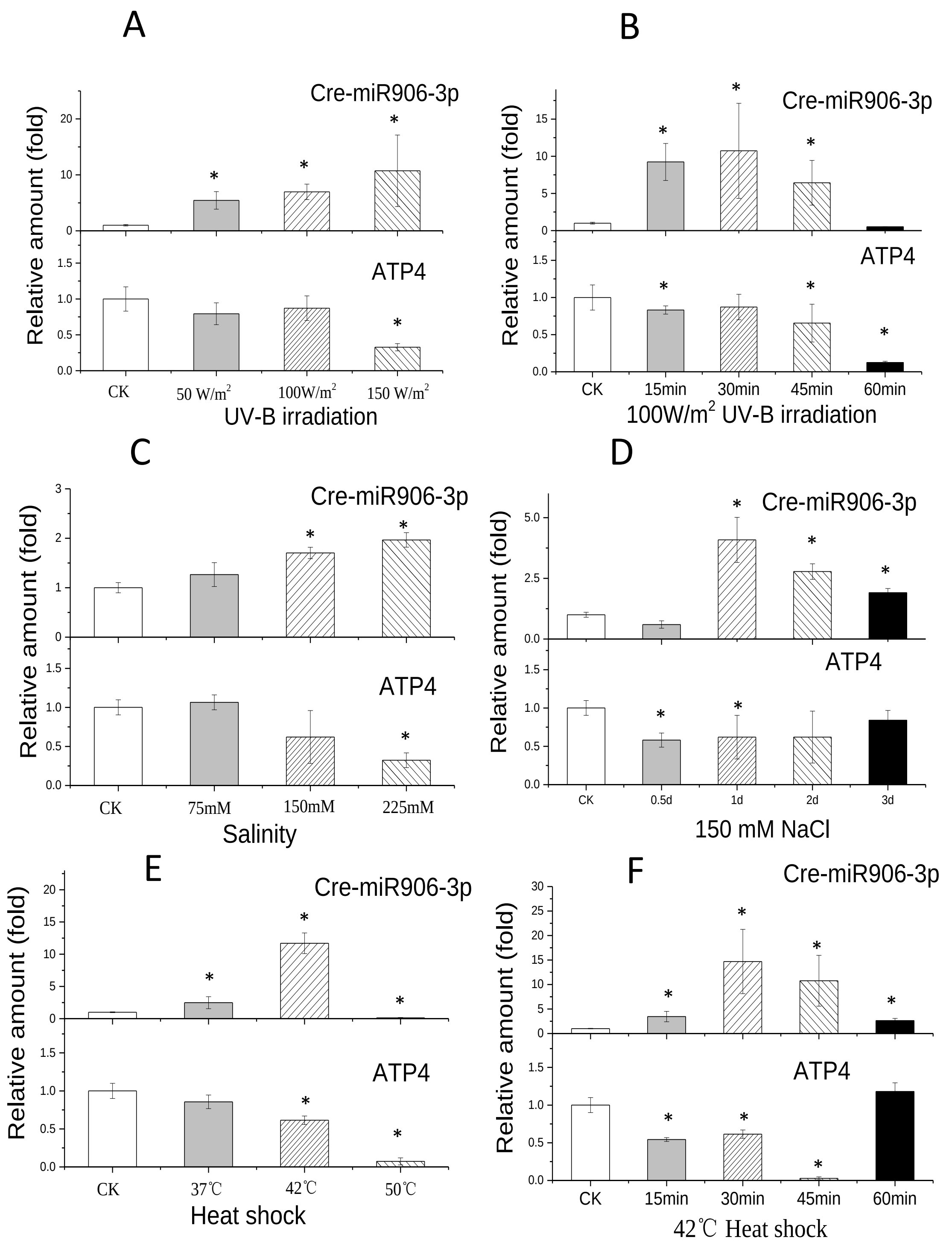

A
Cre-miR906-3p
ATP4
Relative amount (fold)
CK
50 W/m2
100W/m2
150 W/m2
UV-B irradiation
B
Cre-miR906-3p
ATP4
Relative amount (fold)
CK
15min
30min
45min
60min
100W/m2 UV-B irradiation
C
Cre-miR906-3p
ATP4
Relative amount (fold)
CK
75mM
150mM
225mM
Salinity
D
Cre-miR906-3p
ATP4
Relative amount (fold)
CK
0.5d
1d
2d
3d
150 mM NaCl
E
Cre-miR906-3p
ATP4
Relative amount (fold)
CK
37℃
42℃
50℃
Heat shock
F
Cre-miR906-3p
ATP4
Relative amount (fold)
CK
15min
30min
45min
60min
42℃ Heat shock

Fig S2

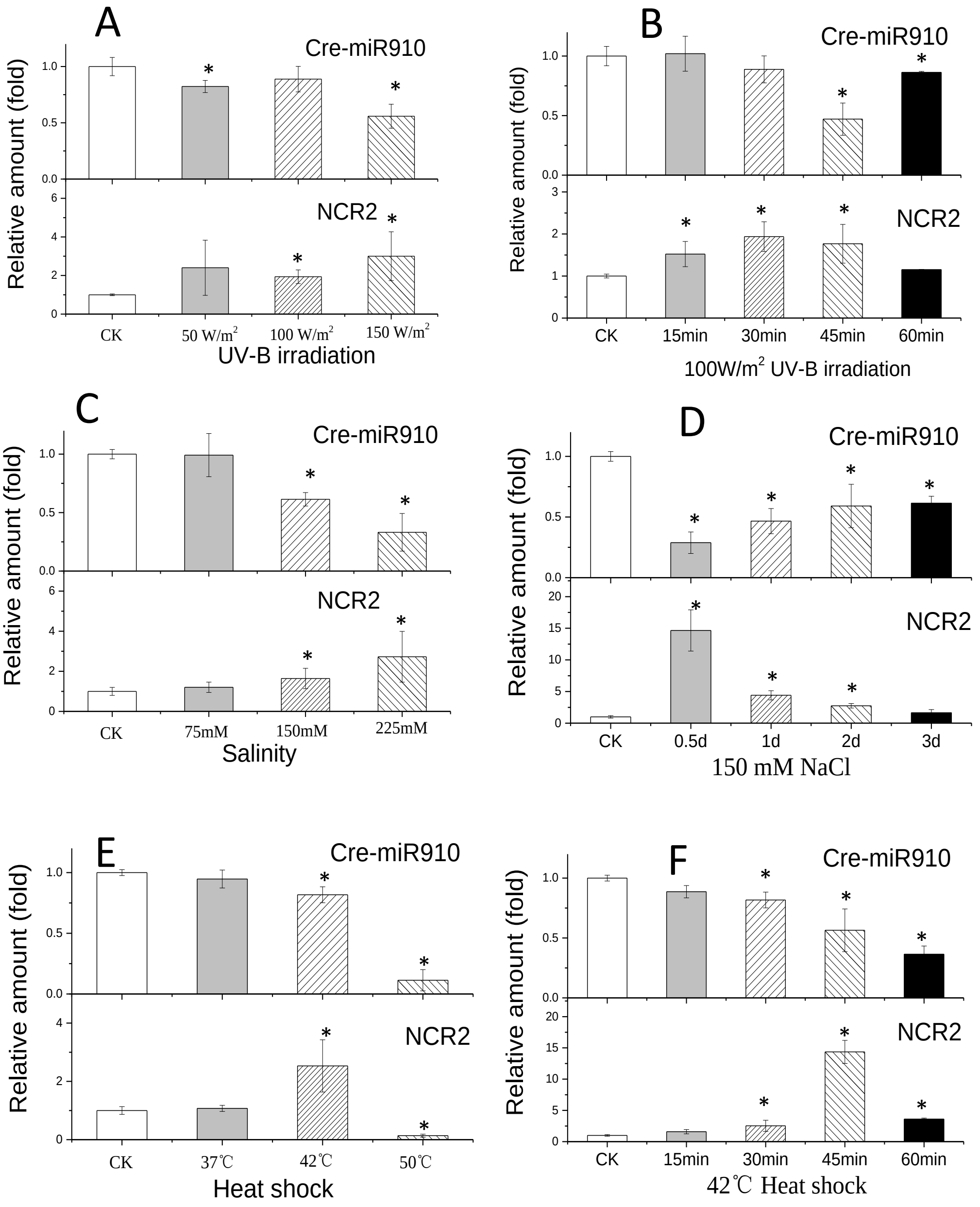

A
Cre-miR910
NCR2
Relative amount (fold)
CK
50 W/m2
100 W/m2
150 W/m2
UV-B irradiation
B
Cre-miR910
NCR2
Relative amount (fold)
CK
15min
30min
45min
60min
100W/m2 UV-B irradiation
C
Cre-miR910
NCR2
Relative amount (fold)
CK
75mM
150mM
225mM
Salinity
D
Cre-miR910
NCR2
Relative amount (fold)
CK
0.5d
1d
2d
3d
150 mM NaCl
E
Cre-miR910
NCR2
Relative amount (fold)
CK
37℃
42℃
50℃
Heat shock
F
Cre-miR910
NCR2
Relative amount (fold)
CK
15min
30min
45min
60min
42℃ Heat shock

Fig S3

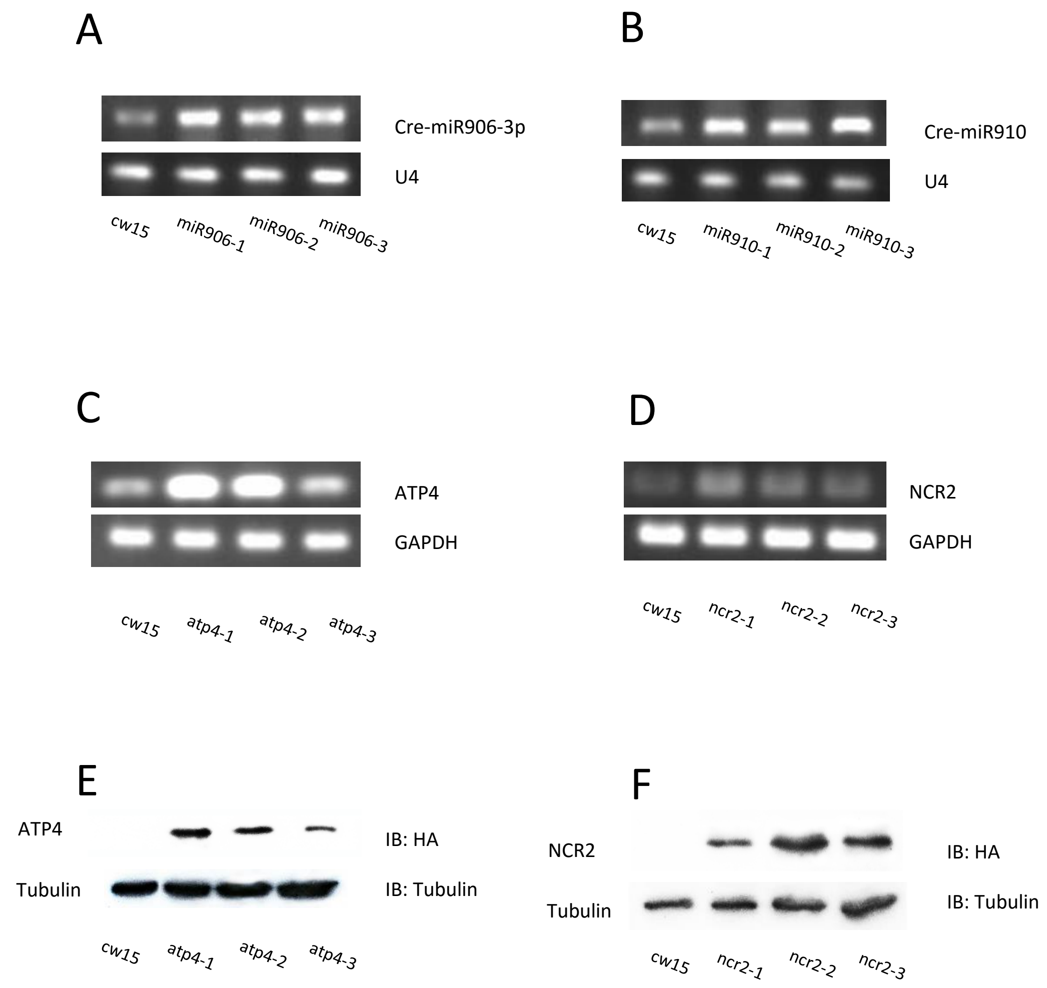
A
Cre-miR906-3p
U4
cw15
miR906-1
miR906-2
miR906-3
B
Cre-miR910
U4
cw15
miR910-1
miR910-2
miR910-3
C
ATP4
GAPDH
cw15
atp4-1
atp4-2
atp4-3
D
NCR2
GAPDH
cw15
ncr2-1
ncr2-2
ncr2-3
E
ATP4
IB: HA
Tubulin
IB: Tubulin
cw15
atp4-1
atp4-2
atp4-3
F
NCR2
IB: HA
Tubulin
IB: Tubulin
cw15
ncr2-1
ncr2-2
ncr2-3

Fig S4

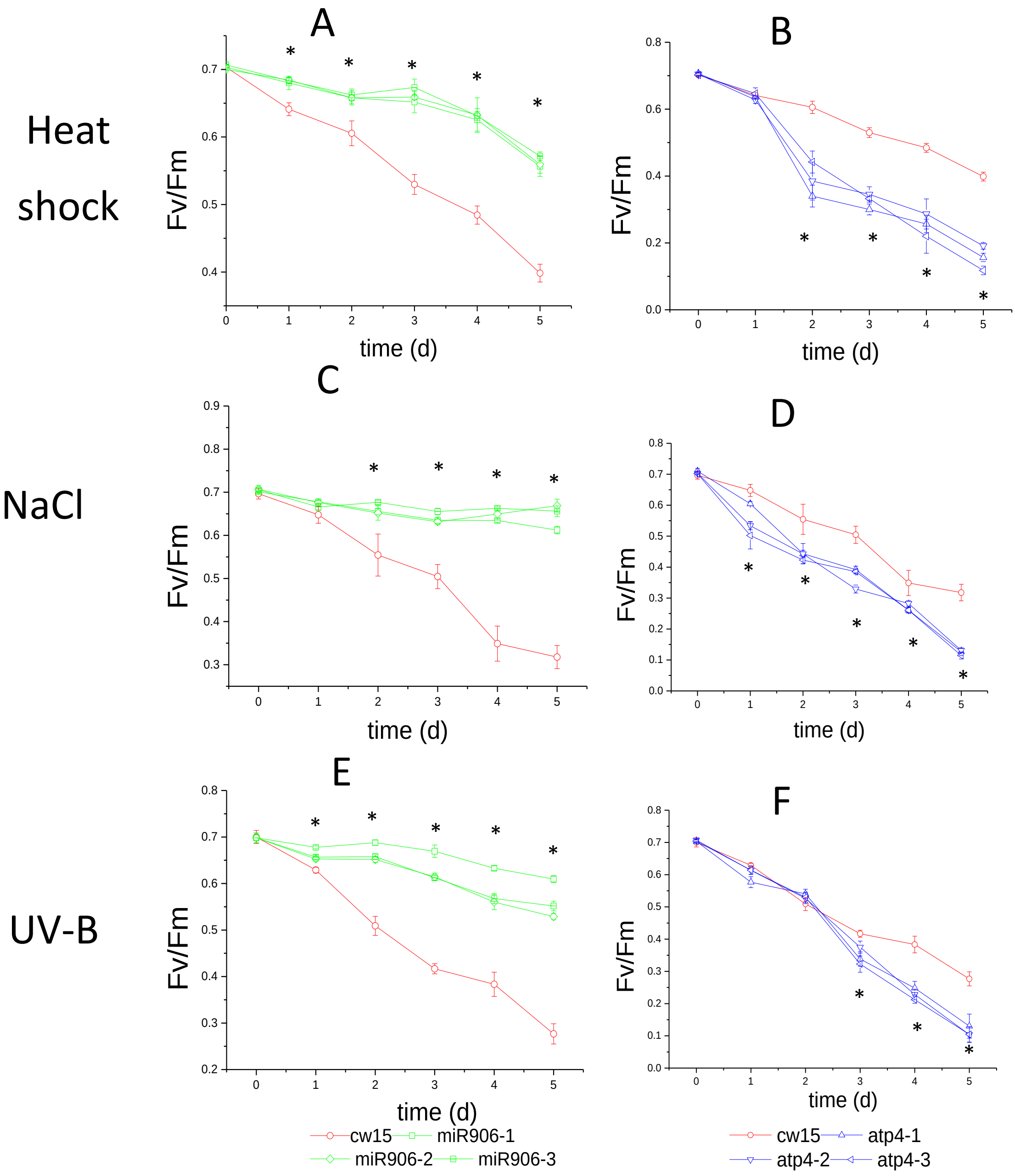
A
B
C
D
E
F
Heat shock
NaCl
UV-B
Fv/Fm
time (d)
cw15
miR906-1
miR906-2
miR906-3
atp4-1
atp4-2
atp4-3

Fig S5

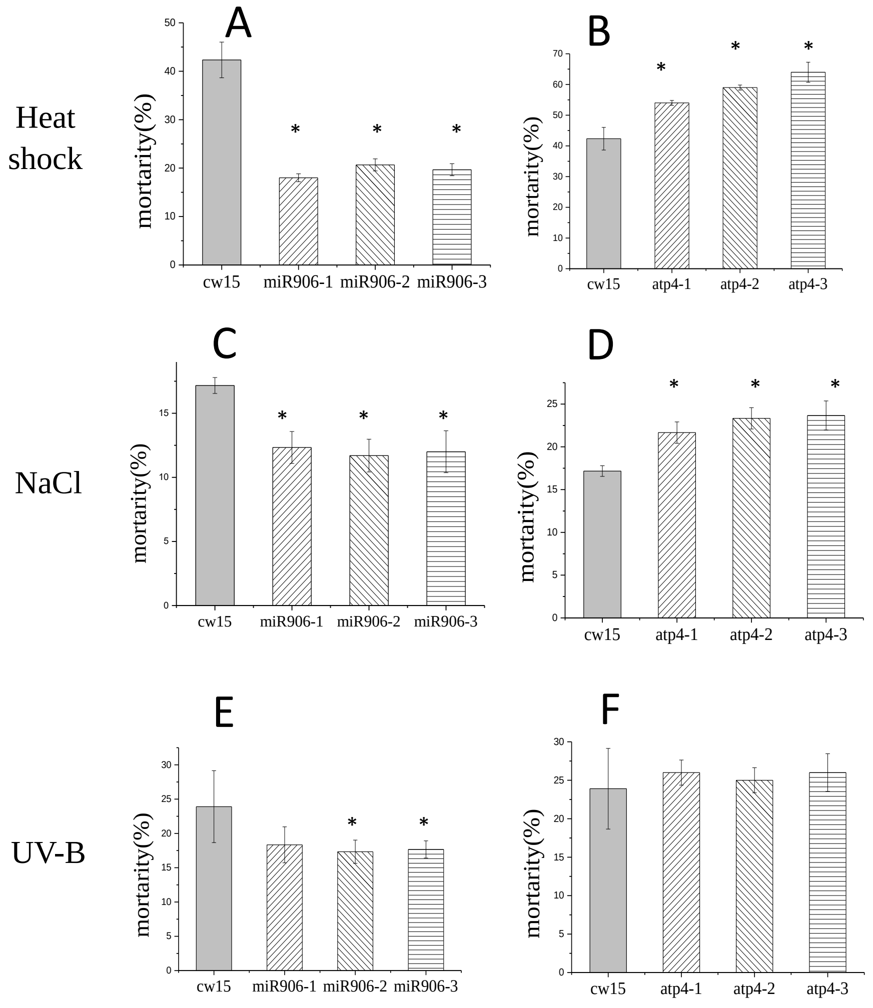
A
B
C
D
E
F
Heat
shock
NaCl
UV-B
mortarity(%)
*
cw15
miR906-1
miR906-2
miR906-3
atp4-1
atp4-2
atp4-3
0
5
10
15
20
25
30
40
50
60
70

Fig S6

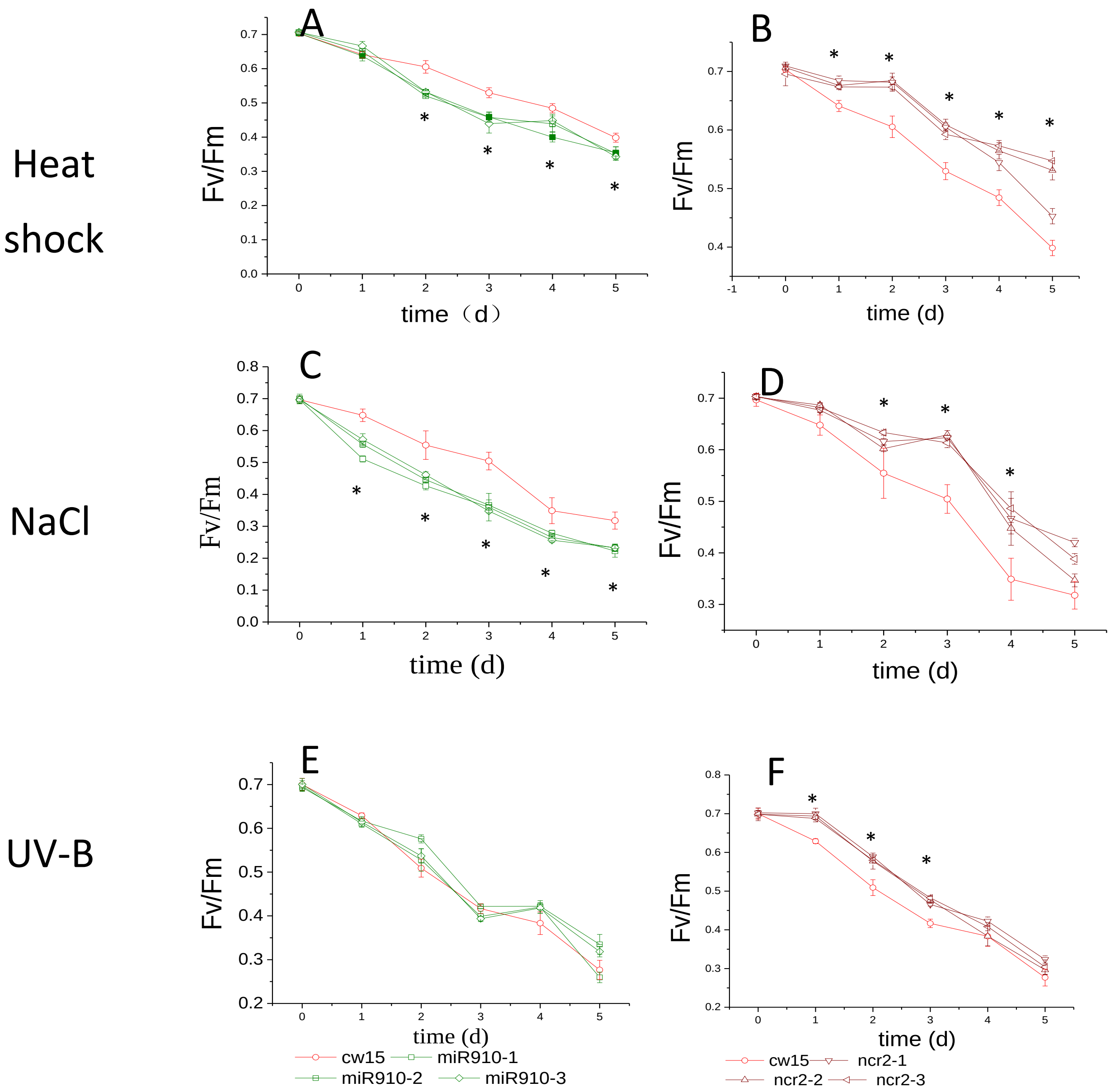
Heat
shock
NaCl
UV-B
A
B
C
D
E
F
Fv/Fm
time（d）
time (d)
cw15
miR910-1
miR910-2
miR910-3
ncr2-1
ncr2-2
ncr2-3

Fig S7

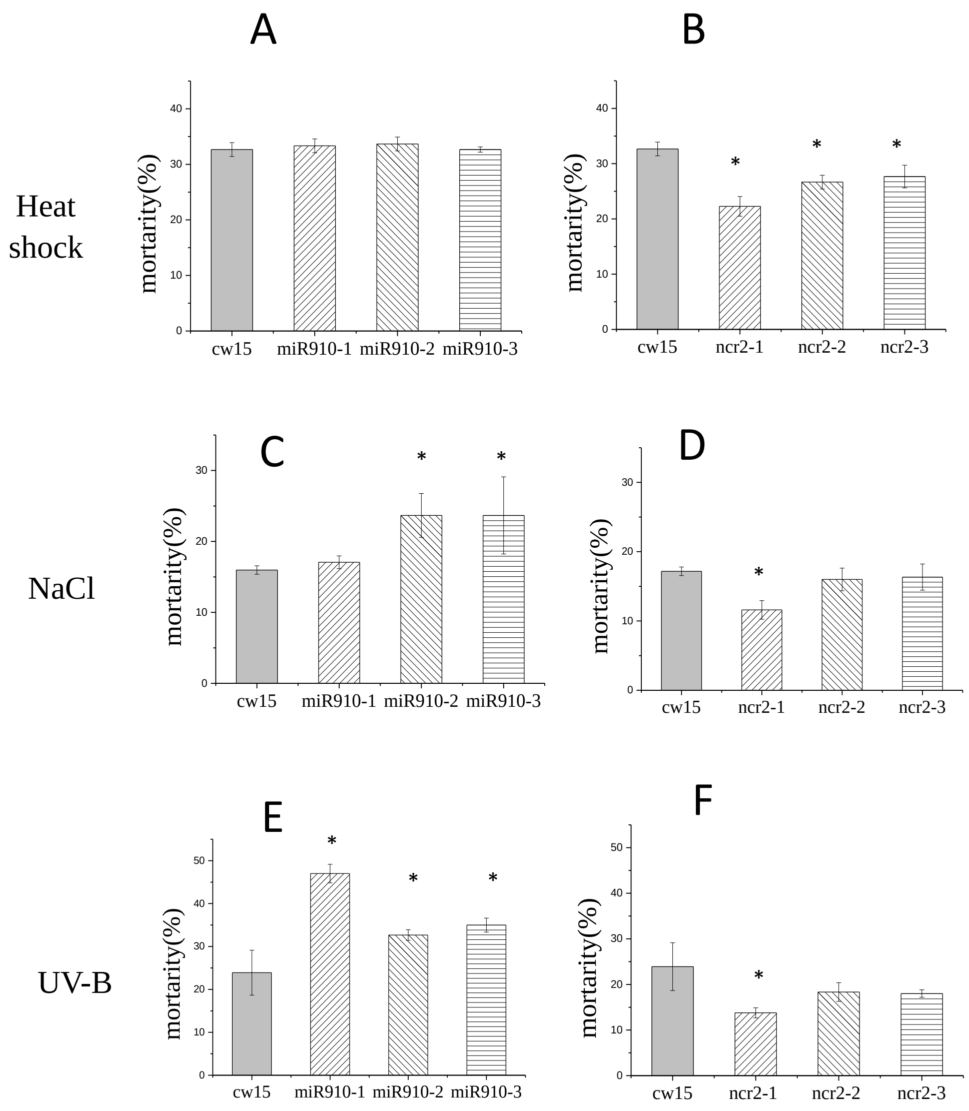
A
B
Heat
shock
mortarity(%)
0
10
20
30
40
cw15
miR910-1
miR910-2
miR910-3
*
ncr2-1
ncr2-2
ncr2-3
C
D
NaCl
E
F
UV-B
50

Tab S1

miRNA name	Accession number/Genbank ID	miRNAs sequence	Forward Primer
cre-miR910	MIMAT0004393/EF495812.1	AGCAGCGUCGGGCUCGACCGC	AGCAGCGTCGGGCTCGACCGC
cre-miR906-3p	MIMAT0004388/EF497926.1	UCCGAUAAAGCUUCCCCCUGC	TCCGATAAAGCTTCCCCCTGC
cre-miR919.2	MIMAT0004399/EF498121.1	UCUCAGGAGGACAUCGCCACU	TCTCAGGAGGACATCGCCACT
cre-miR915	MIMAT0004398/EF498644.1	UGGCAAUAAGGCAAUCGUUGC	TGGCAATAAGGCAATCGTTGC
cre-miR913-5p	MIMAT0004968/EF499286.1	UGCACACUUGCGAGUCCGUGG	TGCACACTTGCGAGTCCGTGG
cre-miR908.2	MIMAT0004390/EF498239.1	UGACGCGUUUGAUAGCAGGAUC	TGACGCGTTTGATAGCAGGATC
cre-miR1167	MIMAT0005434/	GGGGUGUGAUGAUUUGAAAC	GGGGTGTGATGATTTGAAAC
cre-miR1152	MIMAT0005397/EF497494.1	UAAGAAGGUGCGCUGUCUUGA	TAAGAAGGTGCGCTGTCTTGA

Tab S2

Target gene	Forward Primer	Reverse Primer
ATP4	GAACTTCACGAACGTCAGCG	CGCTAGAGAGCTCCACGATG
NCR2	GGTGTCTCTTATGCCGTGTTT	GCCTTCATGGCCTTGAAC
U4	AGTGTCGCAGACTGTGAGG	GGAAGCGTTCCGAAGAA
GAPDH	TGTGAACGAGGGCGACTA	TGCCGAACTTCTGCTCC

Supplement 1： Information of the sequences in this study

The sequence of modified cre-MIR1157 for cre-miR906-3p overexpression:

gcgcagtgttccagctgcagtacGCCTGGGGGAAGCTTTATCGGAtctcgctgatcggcaccatgggg gtggtggtgatcagcgctaTCCGATAAAGCTTCCCCCTGctactgcagccggaacactgccaggagaat t (uppercase letters indicate miRNA*/miRNA sequences).

The sequence of modified cre-MIR1157 for cre-miR910 overexpression:

gcgcagtgttccagctgcagtacGCGGTCGAGCCCGACGGTGCTtctcgctgatcggcaccatggggg tggtggtgatcagcgctaAGCAGCGTCGGGCTCGACCGCtactgcagccggaacactgccaggag aatt (uppercase letters indicate miRNA*/miRNA sequences).

Promoter *HSp70A-RBCS2* sequence:

gacggcggggagctcgctgaggcttgacatgattggtgcgtatgtttgtatgaagctacaggactgatttggcgggc tatgagggcgggggaagctctggaagggccgcgatggggcgcgcggcgtccagaaggcgccatacggcccgctggc ggcacccatccggtataaaagcccgcgaccccgaacggtgacctccactttcagcgacaaacgagcacttatacatacgc gactattctgccgctatacataaccactcagctagcttaagatcccgggcgcgccagaaggagcgcagccaaaccaggat gatgtttgatggggtatttgagcacttgcaacccttatccggaagccccctggcccacaaaggctaggcgccaatgcaagc agttcgcatgcagcccctggagcggtgccctcctgataaaccggccagggggcctatgttctttactttttttacaaga

***HA tail* sequence:**

atgtacccctacgacgtgcccgactacgcctacccctacgacgtgcccgactacgcc

***ATP4* sequence:**

atgctccgcaacgctgctaggcggctcatggccgtgggccagcgcggtattagcacgtctgcgatgaccatggaggagg

tggctgtgccggccggccctaaggagttcaccgaggcctggaacaagaaggcgccctcgcagctgcttgtgcccgagct
gccctcgaacttcacgaacgtcagcgcggacaaccagactcagggagatctgtttcccgtgaacttctacactcccagcag
cgtgctggcggatggcgtgaagaaggacggggtcacgctgccgggcattgatggctacttcggtgtgaaggccaaccac
gttcccgtcatcgctcagctgcgtcctggcatcgtggagctctctagcggcgcggacacggagaagttcttcatcgcgggt
ggctttgccttcgtgcacccgaatggtgtcgcggacatttgcgcgctggaggctggcacgctagaccagttcgaccctgct
gccgttaagtcggcgctggctgccgccaactcggcgcagggccagggcgacgagtacgaccaggccgcgaaccgcg
cggcgctggagctctacgccgccctggactcggcgctggaccagaagtcgtaa

***NCR2* sequence:**

atggacctaaatgttgccttgatagcttgtgtgttcctggtcattgctgcgctagctctgctatctatccgtcgcaaatcgagcg
gtaacgccactgctgtcagcacagccccgccgataactcggacatcaatttctgcagaggaccccagcaagccttgcgtg
cgcatcctctacggcacccagacgggcacggcggtgcgcttctccaagcagcttgccaacgagctgcgcggcaagtatg
gggacagcacggccgtggatgtgcgcgacgtggagacctataaaccagagcggctgggcagcgagaagctggtggtg
atgtgtatggcgacgtatggcgatggcgaacccaccgataacgcagcggtcttctactcgtggctgctcaaggaagcgga
ggcagtcgagaacggcgacaaggagccgtttctgcagggtgtctcttatgccgtgtttgggctgggcaacaagcagtacg
agcacttcaactcggtgggcaagaaggtgttcaaggccatgaaggcgtgcggcgccaccgcgctgtgccgccgcggcg
acggcgacgatgacggcgtcatcgacgacgacttcgagaagtggtgcactgagctgtacgaggcgctggacaagtcca
gcgatctggtcggcaagcgtgccgaccagaacggcgtgcacgcggcgccaccggcgcgtgtagccgcgtacgaagtg
gaggtgctgcgcgggagcggcggcgaggcgccagcctttccctcgggcaccggcaaggacgtgcacagccccttctg
ggccaagattaccaccgtgcgcgagctgcacacccccgcatcggaccgcagctgcgtgcacgtggaggtggatgtcag
cggctcgggcatcacctacgaggcgggcgaccacatcgccatctacgcccgcaacggcgaggcggtggtgagccagg
tggccgagctgctgggctttgacctagaggcccgcattaagctggcgctgccggcggacgcagatgccgcctcaggcct
gccgccgcccttccccggccccgtcaccgtgcgcaccgcgctgtcctacttcgcggacgtgctgtccagcccgcaccgtg

aggcgctgaacgcgctggccagcttcgcggcggaccgggaggaggcggcgcgccttgcgctgctgggctcgccggct

gggaaggccgagtacgcggacttcatcggcaagccgcaccgcagcctgctggaggtgctgcaggccttccccagcgcc

aagcccaccatcggcgccttctttggctgtatcgcgccacgcctgcagcctcgcttctactcaatctcctcctcgcccaagca

gcacccaaacagcgtgcacgtcacgtgcgccgtggtgcgcgacaccatgcccaccggccgcgtgcacgagggcgtgg

cgtcgacgtggctgcaacgccacggcaacggcgcggcggtgcccgtgtttgttcggcactcgcatttccgcctgcccaag

gcggccagcacgccggtggtaatggtgggccccggcaccggcctagcgcccttccgcggcttcctgcaggagcgcgcc

gcgctcaagaactcgggtgcggagctgggccctgcgcacctgttctttggctgccgctcgcgcggcactgactacatctac

cagcaggagctggaggggtatgtggcggacggagtgctgtcgaacctgcacgtggccttctcgcgcgatcagtcctcca

aggactacgtgcagcaccacatagggcgggaagcggccgcgctgtggccgatcatcggggagcagggtgcccacctg

tacgtgtgtggagacgccaagtacatggcgaaggacgtgcacaaggccttcgtggcgctggtggagaagggcaagggc

tgcagcggcacgcaggcggagatgttcgtgaaggagctcacagacgccgggagataccagcgcgatgtctggtaa
